# Diallyl Disulfide Reduces Ethyl Carbamate-Induced Cytotoxicity and Apoptosis in Intestinal and Hepatic Cells

**DOI:** 10.1021/acs.chemrestox.4c00439

**Published:** 2025-03-27

**Authors:** Caroline Andolfato Sanchez, Estefani Maria Treviso, Cecília Cristina de Souza Rocha, Lusânia Maria Greggi Antunes

**Affiliations:** Department of Clinical Analysis, Toxicology Food Science, School of Pharmaceutical Sciences of Ribeirão Preto, University of São Paulo, Av. do Café, Vila Monte Alegre, Ribeirão Preto, SP 14040-903, Brazil

## Abstract

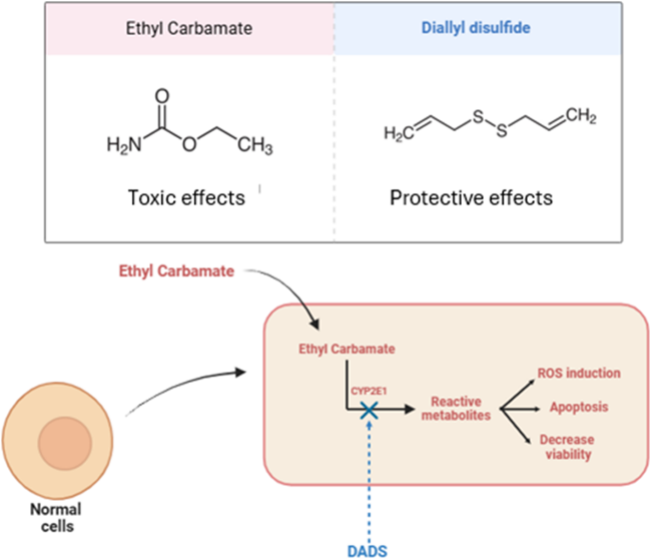

Epidemiological studies indicate that lifestyle and dietary habits are associated with an increasing cancer incidence. Consuming fermented foods and alcoholic beverages and smoking can expose humans to ethyl carbamate (EC), a probable human carcinogen classified as group 2A by the International Agency for Research on Cancer (IARC). Increasing the intake of bioactive compounds can reduce EC-induced toxicity. Diallyl disulfide (DADS), found in garlic, may protect against damage induced by chemical agents and natural compounds. Here, the potential protective effect of DADS against EC was investigated by evaluating EC-induced cytotoxicity, DNA damage, apoptosis, and reactive oxygen species production in colorectal adenocarcinoma (Caco-2) and hepatocarcinoma (HepG2) cells. To this end, resazurin, comet, and annexin V-FITC staining assays and CM-H_2_DCFDA markers were used to evaluate the effect on Caco-2 and HepG2 cells of protocols combining DADS (10–120 μM) and EC (80 mM). The protocols were as follows: (i) cells pretreated with DADS for 2 h and exposed to EC for 24 h; (ii) cells pretreated with DADS for 24 h and exposed to EC for 24 h; (iii) cells simultaneously exposed to DADS and EC for 24 h; (iv) cells exposed to EC for 24 h and treated with DADS for 2 h. EC induced cytotoxicity and apoptosis in Caco-2 and HepG2 cells and oxidative damage in Caco-2 cells. Combined exposure to DADS and EC for 24 h decreased EC-mediated cytotoxicity and apoptosis in both Caco-2 and HepG2 cells. These findings encourage further studies on the mechanisms of action of the combined DADS and EC.

## 1 Introduction

Cancer is one of the leading causes of death worldwide. According to the World Health Organization, colorectal cancer (CRC) is a highly prevalent malignant tumor, ranking as the fourth most common type of cancer and the second leading cause of cancer-related deaths globally.^1^ Hepatic cancer follows as the third leading cause of mortality due to cancer.^1^ The most frequent reasons for rising cancer incidence include risk factors associated with diet and lifestyle, such as smoking, consuming alcohol, and being sedentary.^2^

Epidemiological studies have suggested that the increased number of CRC cases is linked to the consumption of certain fermented foods and beverages.^3^ Although fermentation provides nutritional benefits, it may produce toxic products, including ethyl carbamate (EC), nitrosamines, and biogenic amines,^4^ due to microorganism metabolism.

EC, also known as urethane, is the ethyl ester of carbamic acid. It has historically been used to formulate pesticides and develop cosmetics. It is also applied as an anesthesia solvent. The discovery of its teratogenic and carcinogenic potential^5^ led the International Agency for Research on Cancer (IARC) to classify it as Group 2A.^6^ In addition, the US National Toxicology Program (NTP) lists EC as an animal carcinogen.^7^

Being a natural byproduct of fermentation, EC contaminates fermented foods and beverages.^8^ Major routes of human exposure to EC include inhalation, ingestion, and dermal contact.^9^ In vivo *and* in vitro toxicological studies have demonstrated that EC is carcinogenic and genotoxic to various organs, such as the lungs,^8^ liver, and intestine.^9^ EC is metabolized to toxic compounds like vinyl carbamate epoxide, which is associated with the harmful effects of EC.^10^ Efforts have been made to decrease the EC levels in fermented foods and beverages. Using natural foods rich in bioactive compounds is a promising strategy to reduce the harm caused by EC.^3,11−14^

Diallyl disulfide (DADS), the main organosulfur compound in garlic oil, has attracted attention because it protects against toxicity induced by numerous chemical and natural toxins.^15^ Garlic and its bioactive compounds can mitigate toxicity through mechanisms that reduce oxidative stress, inflammation, apoptosis, and lipid peroxidation.^16,17^ However, no study has evaluated whether DADS can protect against EC effects in vitro.

In this study, we used different protocols to investigate whether DADS protects against EC-induced cytotoxicity, reactive oxygen species (ROS) production, DNA damage, and apoptosis in human adenocarcinoma (Caco-2) and hepatocarcinoma (HepG2) cells. We hypothesized that EC is cytotoxic to Caco-2 and HepG2 cells, primarily through apoptosis-mediated cell death, and that the protective effects of DADS involve reducing ROS production and mitigating EC-induced apoptosis.

## 2 Materials and Methods

### 2.1 Materials

Ethyl carbamate (EC) (CAS 51-79-6); Diallyl disulfide (DADS) (CAS 2179-57-9), resazurin, trypan blue, Tris, dimethyl sulfoxide (DMSO), methylmethanesulfonate (MMS), and hydrogen peroxide (H_2_O_2_) were purchased from Sigma-Aldrich (Saint Louis, MO). Chloromethyl 2′,7′-dichlorodihydrofluorescein diacetate (CM-H_2_DCFDA) was acquired from Invitrogen (Waltham, MA). Dubelcco’s Modified Eagle Medium (DMEM), antibiotic mix (penicillin/streptomycin/neomycin), fetal bovine serum, TrypLE Express, low melting point agarose, normal melting point agarose, and the Annexin V-FITC/Propidium Iodide (PI) assay kit (Catalog No. V13242) were purchased from Thermo Fisher Scientific (Waltham, MA). GelRed dye (Biotium, Hayward, CA) was obtained from Uniscience (São Paulo, SP, Brazil). Doxorubicin hydrochloride was acquired from Eurofarma (São Paulo, SP, Brazil).

### 2.2 Cell Culture

The human adenocarcinoma (Caco-2) and hepatocellular carcinoma (HepG2) cell lines were obtained from the Cell Bank of Rio de Janeiro (BCRJ, Cat. Nos. 0059 and 0103, respectively). The cells were grown in DMEM low glucose culture medium supplemented with 4 mM l-glutamine, 1% v/v antibiotics (penicillin/streptomycin), and 20% v/v (Caco-2 cells) or 10% v/v (HepG2 cells) fetal bovine serum as reported by Bal-Price and Coecke^18^ and maintained in a Forma Series II, Water Jacket CO_2_ Incubator (Thermo Fisher Scientific, Waltham, MA) at 37 °C under 5% CO_2_ and 95% humidity. Assays were conducted between the third and eighth cell passages post-thawing.

### 2.3 Protocol Design

Depending on the assay, the cells were exposed to DADS or EC alone or in combination. Before the assays, DADS was dissolved in DMSO, which provided a 10 mg/mL stock solution and a final-use solution containing 0.25% DMSO. In turn, EC was dissolved in ultrapure water to give a 100 mg/mL solution. For the protocol using DADS or EC alone, the cells were exposed to varying concentrations of DADS (10–120 μM) or EC (10–100 mM) for 24 or 48 h.

To test DADS and EC in combination, the protocols were as follows: (i) the cells were pretreated with DADS (10–120 μM) for 2 h and subsequently exposed to EC (80 mM) for 24 h; (ii) the cells were pretreated with DADS (10–120 μM) for 24 h and subsequently exposed to EC (80 mM) for 24 h; (iii) the cells were simultaneously exposed to DADS (10–120 μM) and EC (80 mM) for 24 h; (iv) the cells were exposed to EC (80 mM) for 24 h and then treated with DADS (10–120 μM) for 2 h.

To evaluate the effects of exposing the cells to EC alone, EC concentrations ranging from 10 to 100 mM were used on the basis of literature studies.^19−22^ To evaluate the effects of treating the cells with DADS alone, DADS concentrations ranging from 10 to 120 μM were employed also on the basis of literature studies.^23−26^ Additionally, these EC and DADS concentrations correspond to in vivo plasma concentrations.^27,28^

### 2.4 Cell Viability Assay

Cell viability was assessed by using the resazurin assay as proposed by Page, Page, and Noel.^29^ Caco-2 or HepG2 cells (1 × 10^4^/well) were seeded in 96-well culture plates and incubated at 37 °C under 5% CO_2_ for 24 h. The cells were treated with 200 μL of DADS alone, exposed to EC alone, or exposed to DADS and EC according to the different protocols described in Section 2.3. MMS (300 μM) was used as the positive control (PC); 0.25% DMSO was employed as the solvent control (SC). Resazurin solution (0.5 mg/mL) was added to each well at 37 °C for 4 h. Fluorescence at 530/590 nm (excitation/emission) was measured on a spectrophotometer (Biotek ELX800, Winooski, VT). Results are presented as the percentage of viable cells relative to the negative control (NC) (DMEM). Cell viability was measured in technical and biological triplicate.

### 2.5 Reactive Oxygen Species Production

Intracellular ROS levels were measured by using the CM-H_2_DCFDA probe and following the manufacturer’s instructions. Caco-2 or HepG2 cells (1 × 10^4^/well) were seeded in 96-well culture plates and incubated at 37 °C under 5% CO_2_ for 24 h. The cells were treated with 200 μL of DADS alone, exposed to EC alone, or exposed to DADS for 24 h and EC for 4 or 24 h. Then, 10 μM CM-H_2_DCFDA was added. Hydrogen peroxide (H_2_O_2_) (1 mM) was used as a positive control for 20 min. Fluorescence at 450/520 nm (excitation/emission) was measured on a Synergy 2 spectrophotometer (BioTek; Winooski, VT). Results are shown as the percentage of intracellular ROS relative to the solvent control, considered to contain 100% ROS. ROS levels were measured in technical and biological triplicate.

### 2.6 Comet Assay

Genotoxicity was analyzed by using the comet assay, according to Tice et al.^30^ Caco-2 or HepG2 cells (5 × 10^4^/well) were seeded in 24-well plates and incubated at 37 °C under 5% CO_2_ for 24 h. The cells were treated with 1 mL of DADS alone (10, 20, or 40 μM), exposed to EC alone (80 mM), or treated with DADS (10, 20, or 40 μM) for 24 h and exposed to EC (80 mM) for 4 or 24 h. MMS (300 μM) was used as a positive control. Cell viability was assessed by trypan blue exclusion on an automatic cell counter Countess (Invitrogen; Carlsbad, CA). All the groups exhibited cell viability greater than 70%. For each sample, 100 nucleoids were analyzed under an AxioStar Plus fluorescence microscope (Carl Zeiss, Axiostar Plus, Jena, Germany) equipped with a camera; a 515–560 nm filter, a 590 nm barrier filter, a 20× objective, and the Comet Assay IV software (Perceptive Instruments; Haverhill, U.K.). Results are presented as DNA percentage in the comet’s tail (tail intensity). The comet assay was carried out in biological triplicate.

### 2.7 Apoptosis Detection by Flow Cytometry

Flow cytometry analysis was performed by using the Dead Cell Apoptosis Kit with Annexin V-FITC and Propidium Iodide (PI) and following the manufacturer’s instructions. Caco-2 or HepG2 cells (5 × 10^5^/well) were seeded in six-well plates and incubated at 37 °C under 5% CO_2_ for 24 h. First, the cells were pretreated with 1 mL of DADS (10, 20, or 40 μM) for 24 h, which was followed by treatment with EC (80 mM) for 24 h. Doxorubicin (2 μM) was used as a positive control. The cells were incubated with Annexin V-FITC for 15 min, mixed with PI (2 μg/mL), and immediately analyzed at 10,000 events per sample. Excitation at 488 nm and emission at 530/575 nm (or equivalent) were read on a BD LSR Fortessa Flow Cytometer (BD Biosciences, San Jose, CA). Apoptotic and necrotic cells were measured in biological triplicate.

### 2.8 Statistical Analysis

Statistical analysis and IC_50_ calculations were performed by using GraphPad Prism 8.0 (Boston, MA). All the tests with *p* < 0.05 were considered statistically significant. The results are expressed as the mean ± standard deviation. For all the assays, data normality was verified by employing the Shapiro-Wilk test. The cell viability assay data obtained from treatment with DADS alone or exposure to EC alone were subjected to analysis of variance (One-Way ANOVA) followed by Tukey’s multiple comparison post-test. In the assays involving cells submitted to treatment with DADS alone or exposure to EC alone, the data obtained from the positive control assays were subjected to Student’s *t*-test compared to the solvent control. For combined exposure to DADS and EC, the data obtained from the assays involving cell exposure to EC alone were subjected to Student’s *t*-test compared to the negative control, and the data obtained from the assays involving cell exposure to DADS and EC were subjected to analysis of variance (One-Way ANOVA) followed by Dunnett’s post-test compared to the data obtained from the assays involving exposure to EC alone.

## 3 Results

### 3.1 Protocol for Cell Exposure to Either Ethyl Carbamate or Diallyl Disulfide

#### 3.1.1 Cytotoxicity of Ethyl Carbamate

**Figure 1:**
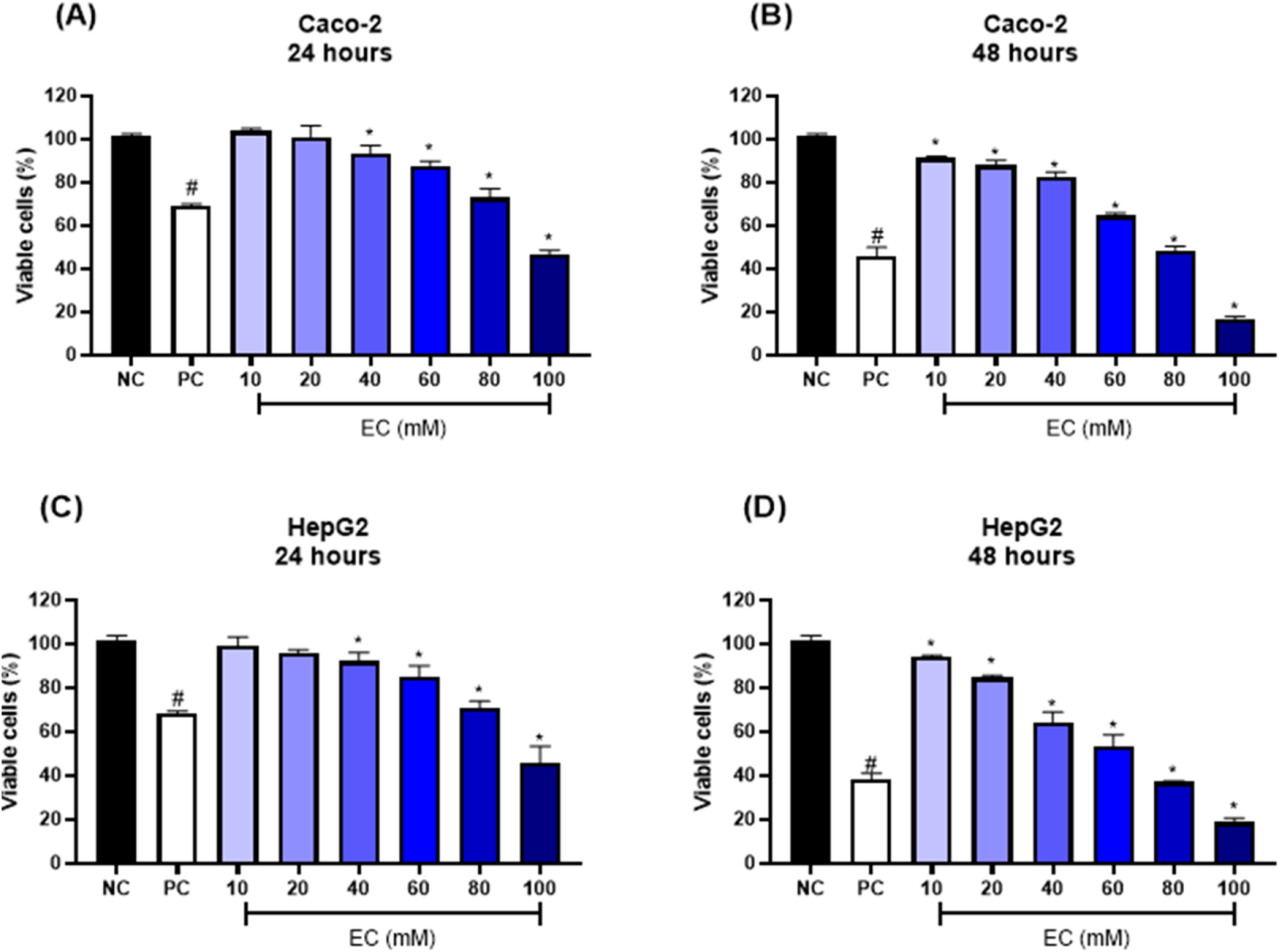
Viability (%) of (A, B) colorectal adenocarcinoma (Caco-2) and (C, D) hepatocarcinoma (HepG2) cells after exposure to ethyl carbamate (EC) (10:100 mM) for 24 or 48 h. NC: negative control (Dubelcco’s Modified Eagle Medium); PC: positive control (300 μM methylmethanesulfonate). Results are expressed as the mean ± standard deviation (*n* = 3). *p < 0.05; One-Way ANOVA vs negative control; ^#^*p* < 0.05 (Student’s *t*-Test) vs negative control.

Exposure to EC decreased Caco-2 and HepG2 cell viability in a concentration- and time-dependent manner (Figure 1). IC_50_ was 98 mM at 24 h, 70.7 mM at 48 h for Caco-2 cells, 97.7 mM at 24 h, and 56 mM at 48 h for HepG2 cells (Table 1).

**Table 1:** IC_50_ Values for Ethyl Carbamate against Colorectal Adenocarcinoma (Caco-2) or Hepatocarcinoma (HepG2) Cells

cells	IC_50_ at 24 h (mM)	IC_50_ at 48 h (mM)
Caco-2	98.0	70.7
HepG2	97.7	56.0

#### 3.1.2 Cytotoxicity of Diallyl Disulfide

**Figure 2:**
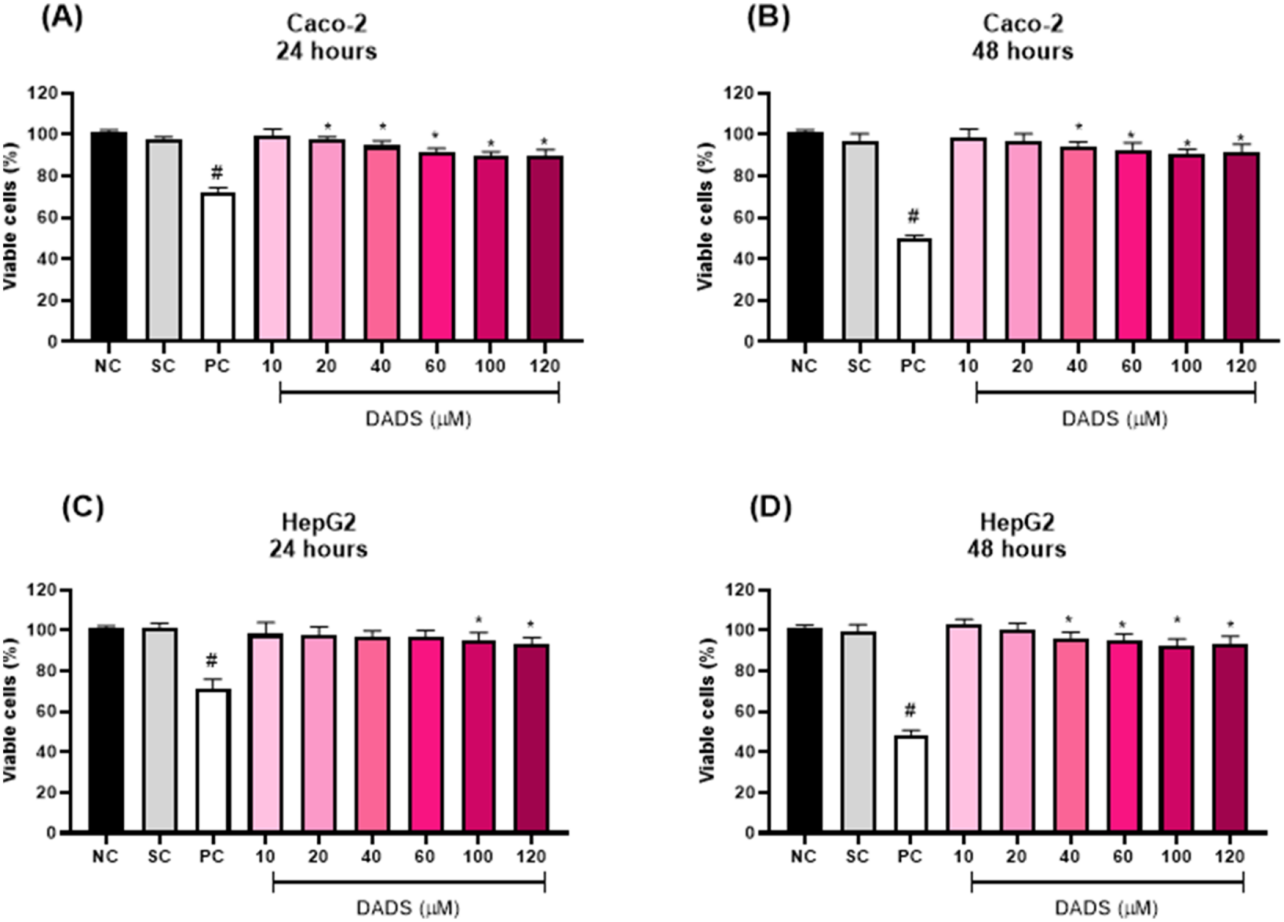
Viability (%) of (A, B) colorectal adenocarcinoma (Caco-2) and (C, D) hepatocarcinoma (HepG2) cells after treatment with diallyl disulfide (DADS) (10–120 μM) for 24 or 48 h. NC: negative control (Dubelcco’s Modified Eagle Medium); PC: positive control (300 μM methylmethanesulfonate); SC: solvent control (0.25% dimethyl sulfoxide). Results are expressed as the mean ± standard deviation (*n* = 3). *p < 0.05; One-Way ANOVA vs solvent control; ^#^*p* < 0.05 (Student’s *t*-Test) vs negative control.

DADS decreased the Caco-2 and HepG2 cell viability in a concentration-independent manner (Figure 2). Compared to the solvent control, Caco-2 cell viability was significantly reduced starting from 20 μM DADS after 24 h (Figure 2A) and 40 μM DADS after 48 h (Figure 2B). In the case of HepG2 cells, higher DADS concentrations (100–120 μM) significantly decreased cell viability compared to the solvent control after 24 h (Figure 2C). At 48 h, DADS at 40–120 μM significantly reduced HepG2 cell viability compared to the solvent control (Figure 2D). We were not able to determine IC_50_ for DADS because none of the tested concentrations decreased Caco-2 or HepG2 cell viability by 50% compared to that of the negative control.

### 3.2 Protocol for Cell Exposure to Ethyl Carbamate Combined with Diallyl Disulfide

On the basis of the results obtained for the cell viability assays conducted with the cells treated with DADS alone, we selected all of the tested DADS concentrations (10–120 μM). On the basis of the results obtained for the cell viability assays involving exposure to EC alone, we selected 80 mM EC to ensure minimal cytotoxicity, according to the literature.^19−22^

#### 3.2.1 Viability of Cells Pretreated with Diallyl Disulfide for 2 h Followed by Exposure to Ethyl Carbamate for 24 h

**Figure 3:**
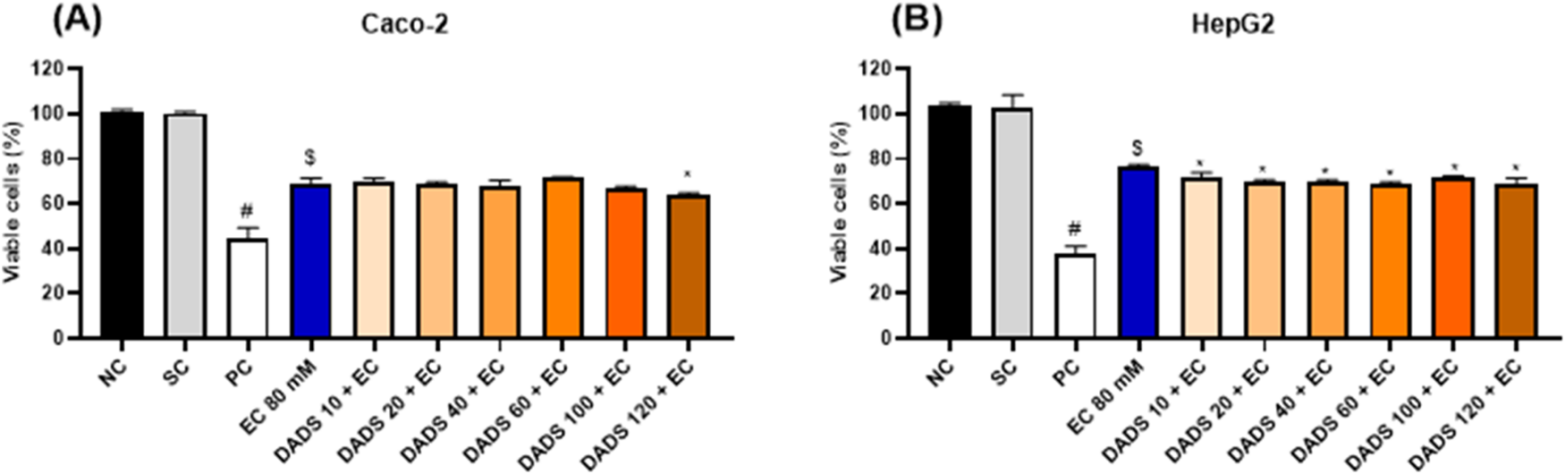
Viability (%) of (A) colorectal adenocarcinoma (Caco-2) and (B) hepatocarcinoma (HepG2) cells after pretreatment with diallyl disulfide (DADS) (10–120 μM) for 2 h followed by exposure to ethyl carbamate (EC) (80 mM) for 24 h. NC: negative control (Dubelcco’s Modified Eagle Medium); PC: positive control (300 μM methylmethanesulfonate); SC: solvent control (0.25% dimethyl sulfoxide). Results are expressed as the mean ± standard deviation (*n* = 3). ^$^*p* < 0.05 (Student’s *t*-Test) vs solvent control. *p < 0.05; One-Way ANOVA vs 80 mM EC; ^#^*p* < 0.05 (Student’s *t*-Test) vs negative control.

Pretreatment with DADS for 2 h followed by exposure to EC for 24 h did not increase Caco-2 or HepG2 cell viability compared to exposure to EC alone for 24 h (Figure 3). In Caco-2 cells, pretreatment with the highest DADS concentration (120 μM) for 2 h followed by exposure to EC for 24 h significantly decreased cell viability compared with exposure to EC alone for 24 h (Figure 3A). In HepG2 cells, pretreatment with DADS for 2 h followed by exposure to EC for 24 h significantly decreased cell viability compared with exposure to EC alone for 24 h irrespective of the DADS concentration (Figure 3B).

#### 3.2.2 Viability of Cells Pretreated with Diallyl Disulfide for 24 h Followed by Exposure to Ethyl Carbamate for 24 h

**Figure 4:**
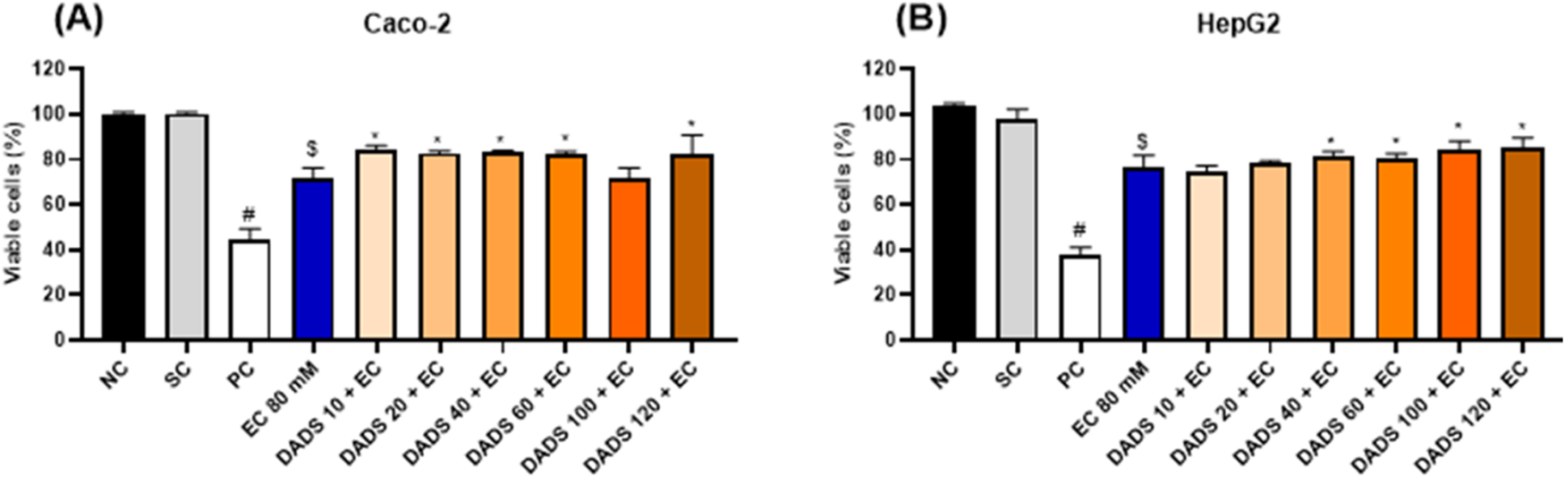
Viability (%) of (A) colorectal adenocarcinoma (Caco-2) and (B) hepatocarcinoma (HepG2) cells after pretreatment with diallyl disulfide (DADS) (10:120 μM) for 24 h followed by exposure to ethyl carbamate (EC) (80 mM) for 24 h. NC: negative control (Dubelcco’s Modified Eagle Medium); PC: positive control (300 μM methylmethanesulfonate); SC: solvent control (0.25% dimethyl sulfoxide). Results are expressed as the mean ± standard deviation (*n* = 3). ^$^*p* < 0.05 (Student’s *t*-Test) vs solvent control. *p < 0.05; One-Way ANOVA vs 80 mM EC; ^#^*p* < 0.05 (Student’s *t*-Test) vs negative control.

Pretreatment with DADS for 24 h followed by exposure to EC for 24 h significantly increased Caco-2 cell viability compared to exposure to EC alone for 24 h (Figure 4A).

Compared to HepG2 cells exposed to EC alone for 24 h, pretreatment with DADS for 24 h and exposure to EC for 24 h significantly increased HepG2 cell viability by 7.2, 6.6, 10.5, and 11.7% at 40, 60, 100, and 120 μM DADS, respectively (Figure 4B).

#### 3.2.3 Viability of Cells Simultaneously Exposed to Diallyl Disulfide and Ethyl Carbamate for 24 h

**Figure 5:**
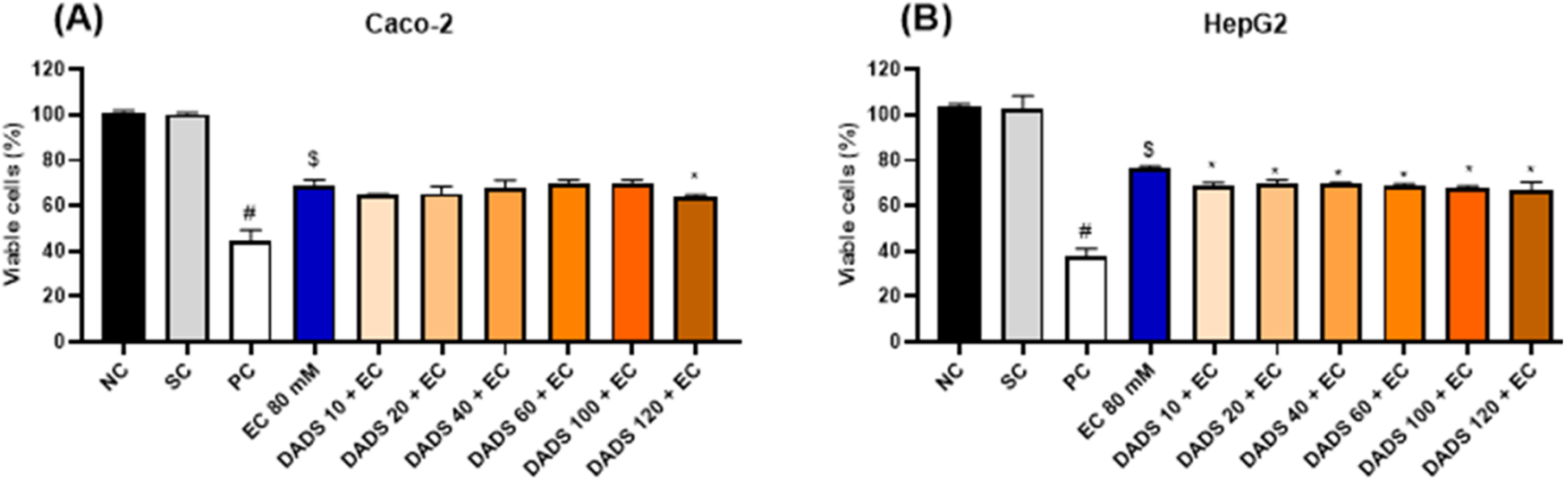
Viability (%) of (A) colorectal adenocarcinoma (Caco-2) and (B) hepatocarcinoma (HepG2) cells after simultaneous exposure to diallyl disulfide (DADS) (10–120 μM) and ethyl carbamate (EC) (80 mM) for 24 h. NC: negative control (Dubelcco’s Modified Eagle Medium); PC: positive control (300 μM methylmethanesulfonate); SC: solvent control (0.25% dimethyl sulfoxide). Results are expressed as the mean ± standard deviation (*n* = 3). ^$^*p* < 0.05 (Student’s *t*-Test) vs solvent control. *p < 0.05; One-Way ANOVA vs 80 mM EC; ^#^*p* < 0.05 (Student’s *t*-Test) vs negative control.

Simultaneous exposure to DADS and EC for 24 h did not increase Caco-2 or HepG2 cell viability compared to exposure to EC alone for 24 h (Figure 5). Instead, simultaneous exposure to 120 μM DADS and EC for 24 h decreased Caco-2 cell viability compared to exposure to EC alone for 24 h (Figure 5A), and simultaneous exposure to EC and DADS at any of the tested concentrations for 24 h decreased HepG2 cell viability (Figure 5B) compared with exposure to EC alone for 24 h.

#### 3.2.4 Viability of Cells Exposed to Ethyl Carbamate for 24 h Followed by Treatment with Diallyl Disulfide for 2 h

**Figure 6:**
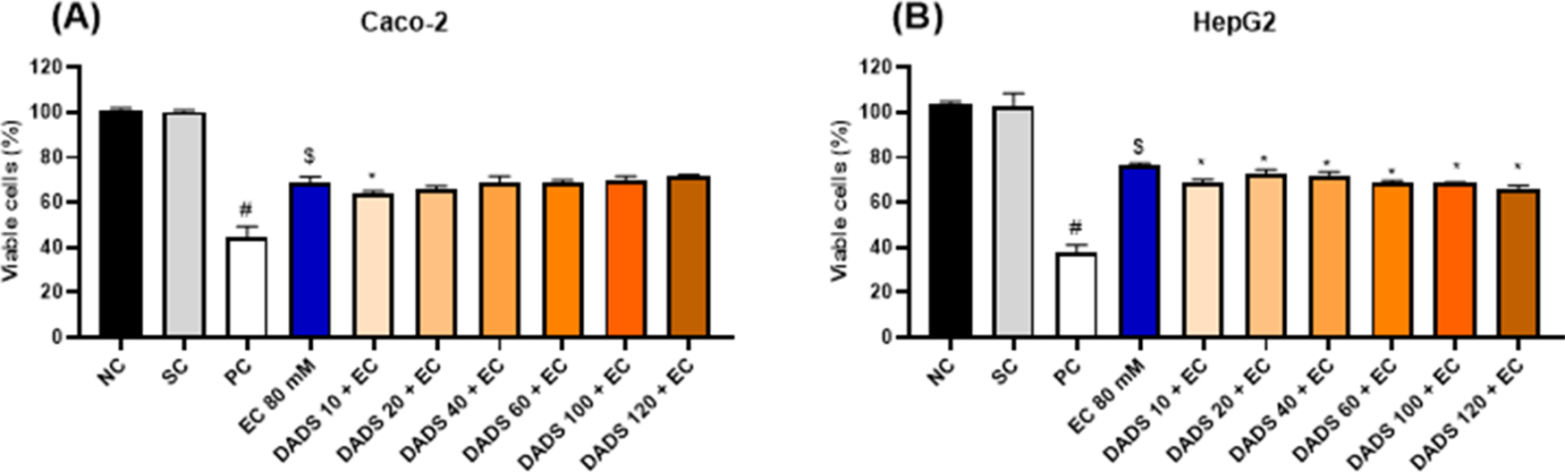
Viability (%) of (A) colorectal adenocarcinoma (Caco-2) and (B) hepatocarcinoma (HepG2) cells after exposure to ethyl carbamate (EC) (80 mM) for 24 h followed by treatment with diallyl disulfide (DADS) (10:120 μM) for 2 h. NC: negative control (Dubelcco’s Modified Eagle Medium); PC: positive control (300 μM methylmethanesulfonate); SC: solvent control (0.25% dimethyl sulfoxide). Results are expressed as the mean ± standard deviation (*n* = 3). ^$^*p* < 0.05 (Student’s *t*-Test) vs solvent control. *p < 0.05; One-Way ANOVA vs 80 mM EC; ^#^*p* < 0.05 (Student’s *t*-Test) vs negative control.

Exposure to EC for 24 h followed by treatment with DADS for 2 h did not inhibit the EC-induced cytotoxicity in Caco-2 cells. Indeed, exposure to EC for 24 h followed by treatment with 10 μM DADS significantly decreased Caco-2 cell viability (Figure 6A). In turn, exposure to EC for 24 h followed by treatment with any of the tested DADS concentrations for 2 h significantly decreased HepG2 cell viability compared to exposure to EC alone for 24 h (Figure 6B).

Bearing in mind the results presented above and the objectives of this study, which aims to evaluate the protective effect of DADS against the harmful effects of EC, we decided to use protocol (ii), that is, pretreatment with DADS for 24 h followed by exposure to EC for 24 h, in the next assays. We selected the three lowest tested DADS concentrations (10, 20, and 40 μM) on the basis of the results achieved for the cells treated with DADS alone: at 10–40 μM, DADS did not decrease HepG2 cell viability compared to the solvent control, and these concentrations correspond to DADS concentrations in plasma in vivo.^27^ In addition, on the basis of the results achieved during protocol (ii), DADS at 10–40 μM associated with EC exerted a protective effect compared to cells exposed to EC alone for 24 h.

### 3.3 Pretreatment of Cells with Diallyl Disulfide for 24 h Followed by Exposure to Ethyl Carbamate Does Not Increase DNA Damage

**Figure 7:**
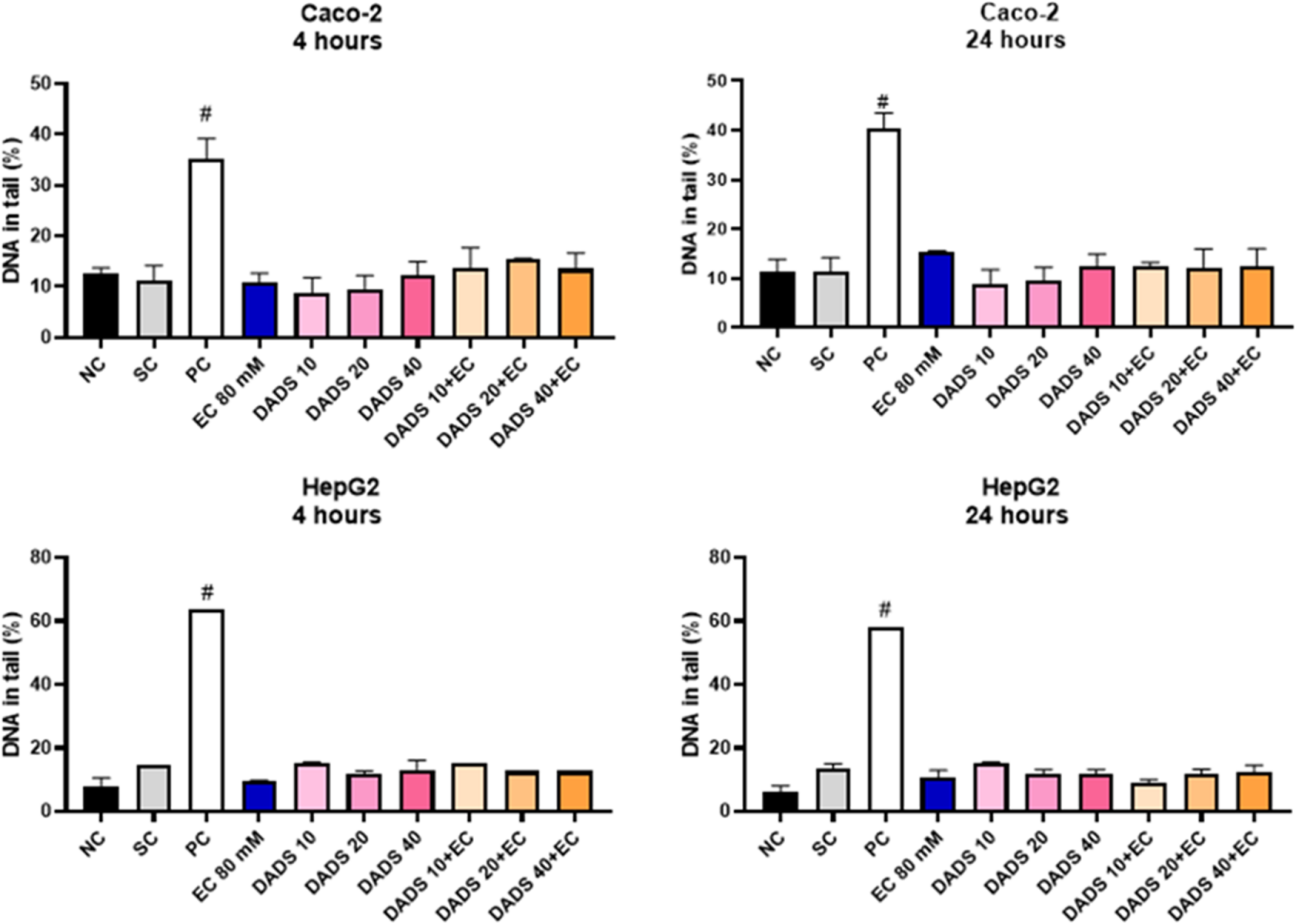
Percentage of DNA in the tail of (A, B) colorectal adenocarcinoma (Caco-2) and (C, D) hepatocarcinoma (HepG2) cells after pretreatment with diallyl disulfide (DADS) for 24 h, followed by exposure to ethyl carbamate (EC) (80 mM) for 4 or 24 h. NC: negative control (Dubelcco’s Modified Eagle Medium); PC: positive control (300 μM methylmethanesulfonate); SC: solvent control (0.25% dimethyl sulfoxide). Results are expressed as the mean ± standard deviation (*n* = 3). ^$^*p* < 0.05 (Student’s *t*-Test) vs solvent control. *p < 0.05; One-Way ANOVA vs 80 mM EC; ^#^*p* < 0.05 (Student’s *t*-Test) vs negative control.

Regarding DNA damage, assessment of the protective effects of DADS against EC-induced cytotoxicity by the comet assay after cells were pretreated with DADS for 24 h and exposed to EC for 4 or 24 h showed that DNA was not damaged in Caco-2 (Figure 7A) or HepG2 cells (Figure 7B) compared with the solvent control.

### 3.4 Pretreatment with Diallyl Disulfide Reduces Reactive Oxygen Species Production Induced by Exposure to Ethyl Carbamate for 24 h

Considering that DADS mitigates the EC-induced reduction in cell viability induced by EC, we determined whether the EC-induced cytotoxicity is related to increased ROS production and whether DADS protects cells by inhibiting ROS production.

**Figure 8:**
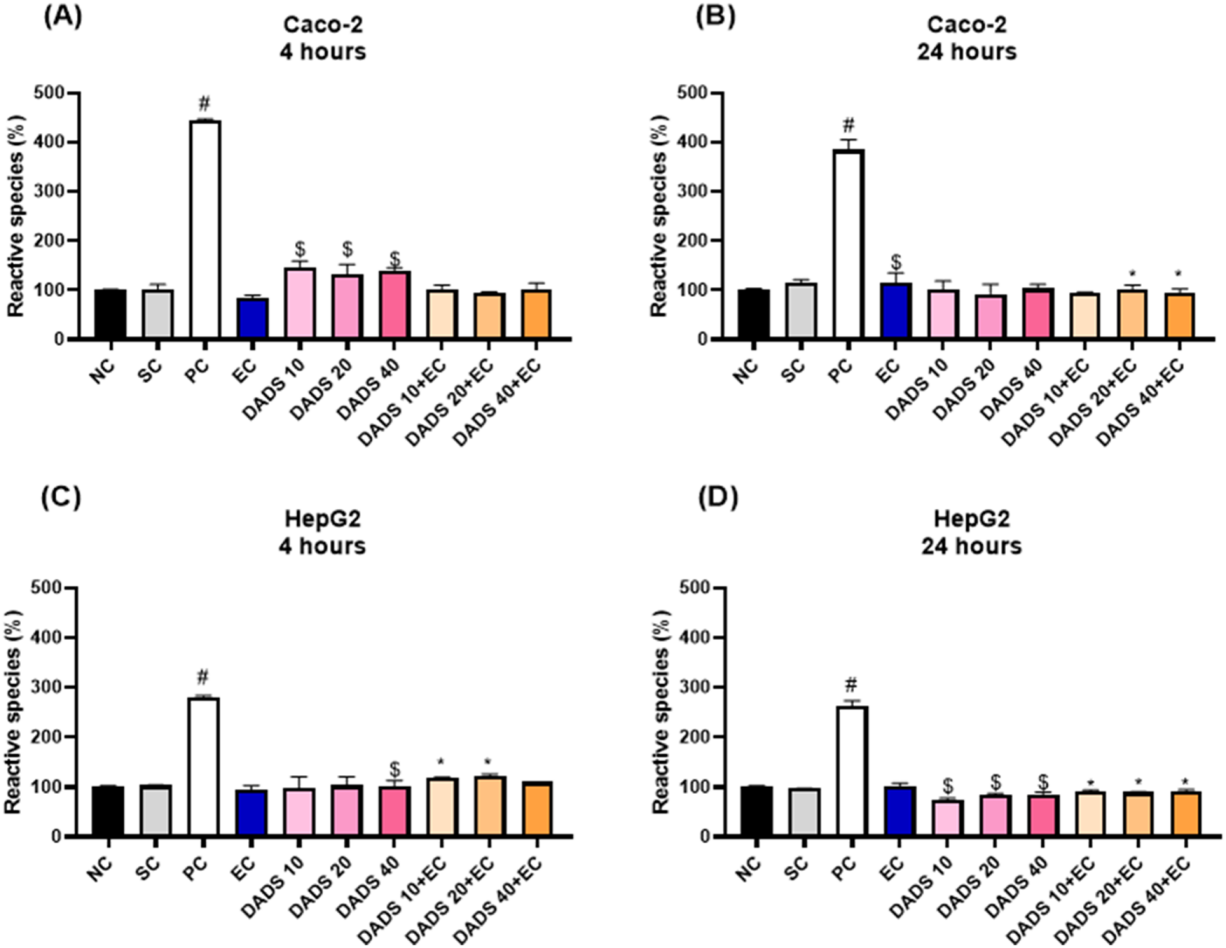
Intracellular reactive oxygen species (ROS) production in (A, B) colorectal adenocarcinoma (Caco-2) and (C, D) hepatocarcinoma (HepG2) cells after treatment with diallyl disulfide (DADS) for 24 h followed by exposure to ethyl carbamate (EC) (80 mM) for 4 or 24 h. NC: negative control (Dubelcco’s Modified Eagle Medium); PC: positive control (300 μM methylmethanesulfonate); SC: solvent control (0.25% dimethyl sulfoxide). Results are expressed as the mean ± standard deviation (*n* = 3). ^$^*p* < 0.05 (Student’s *t*-Test) vs solvent control. *p < 0.05; One-Way ANOVA vs 80 mM EC; ^#^*p* < 0.05 (Student’s *t*-Test) vs negative control.

In Caco-2 cells, exposure to EC alone for 4 h or pretreatment with DADS for 24 h followed by exposure to EC for 4 h did not increase ROS production, whereas pretreatment with DADS alone increased ROS production compared with the solvent control (Figure 8A). As for exposure to EC alone for 24 h, it increased ROS production by 23.6% compared with the solvent control (Figure 8B). In turn, pretreatment with DADS at 20 or 40 μM for 24 h followed by exposure to EC for 24 h decreased ROS production compared with exposure to EC alone for 24 h (Figure 8B).

In HepG2 cells, exposure to EC alone for 4 or 24 h did not increase the ROS production (Figure 8C,D, respectively). However, treatment with DADS alone decreased ROS production compared to the solvent control. In turn, pretreatment with DADS for 24 h followed by exposure to EC for 24 h decreased ROS production compared to exposure to EC alone for 24 h.

### 3.5 Diallyl Disulfide Protects against Ethyl Carbamate-Induced Apoptosis and Necrosis

**Figure 9:**
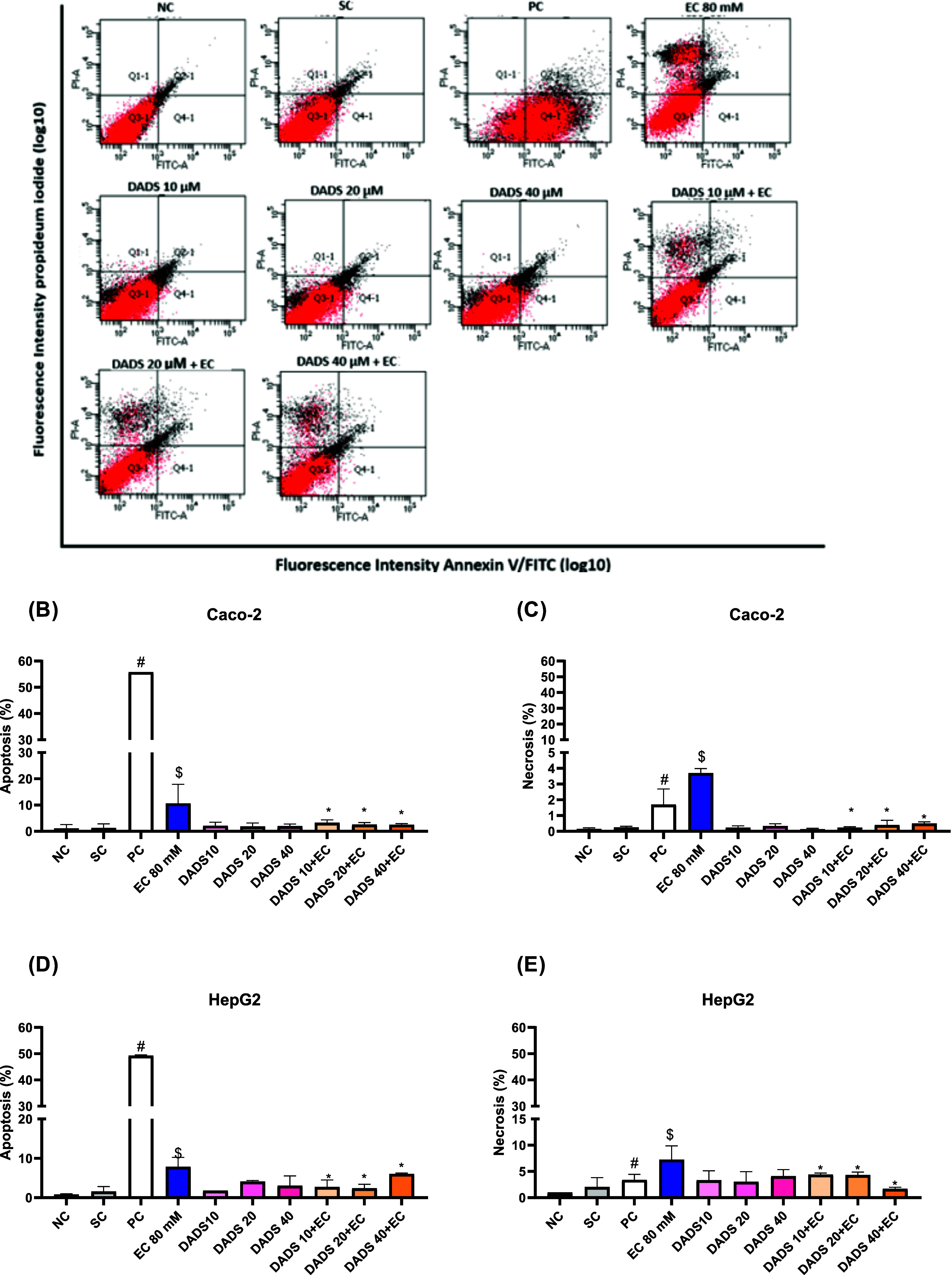
Cell death profile of (A–C) colorectal adenocarcinoma (Caco-2) and (D, E) hepatocarcinoma (HepG2) cells after pretreatment with diallyl disulfide (DADS) for 24 h followed by exposure to ethyl carbamate (EC) for 24 h, analyzed by staining with Annexin V-FITC/PI. (A) Distribution of cells stained with Annexin V-FITC/PI. Lower left quadrant: cells not stained with any of the dyes. Lower right quadrant: cells stained with Annexin V-FITC. Upper left quadrant: cells stained with PI. Upper right quadrant: cells stained with both dyes. (B, D) Percentage of apoptotic cells (Q2+Q4). (C, E) Percentage of necrotic cells (Q1). NC: negative control (Dubelcco’s Modified Eagle Medium); SC: solvent control (0.25% DMSO); PC: positive control (2 μM Doxorubicin). Mean ± standard deviation (*n* = 3). ^#^*p* < 0.05 represents a significant difference compared to the negative control (Student’s *t*-Test), ^$^*p* < 0.05 represents a significant difference compared to the solvent control (Student’s *t*-Test), *p < 0.05 represents a significant difference compared to 80 mM EC (One-Way ANOVA-Dunnett’s).

To assess whether the decreased cell viability induced by EC and the protective effect of DADS involve cell death, we investigated apoptosis and necrosis by using flow cytometry (Figure 9).

Figure 9A shows the distribution pattern of the cells stained with Annexin V-FITC/PI for all of the assays.

Analysis of apoptotic and necrotic cells (Figure 9B–E) revealed that the solvent control (0.25% DMSO) did not induce apoptosis or necrosis in Caco-2 or HepG2 cells, resembling the results achieved with the negative control (culture medium). On the other hand, the positive control (2 μM doxorubicin) effectively induced apoptosis and necrosis in Caco-2 and HepG2 cells compared to the negative control.

For Caco-2 cells, treatment with DADS alone did not induce apoptosis or necrosis compared with the solvent control (Figure 9B,C, respectively). In contrast, exposure to EC alone significantly increased the percentage of apoptotic and necrotic Caco-2 cells compared to the negative control, with apoptosis increasing by 20% (Figure 9B) and necrosis increasing by 5% (Figure 9C). As for Caco-2 cells treated with DADS for 24 h followed by exposure to EC for 24 h, apoptosis and necrosis decreased at all of the tested DADS concentrations, by approximately 70%, compared to exposure to EC alone (Figure 9B,C, respectively).

In the case of HepG2 cells, treatment with DADS alone did not increase the number of apoptotic or necrotic cells compared to the solvent control (Figure 9D,E, respectively). Exposure to EC alone increased the percentage of apoptotic and necrotic Hep2G cells percentage by 10% compared to the negative control. Furthermore, treating Hep2G cells with 10, 20, or 40 μM DADS for 24 h and exposing them to EC for 24 h reduced the percentage of apoptotic and necrotic cells compared with exposure to EC alone (Figure 9D,E, respectively).

## 4 Discussion

The rising incidence and mortality rates of colorectal cancer (CRC) and hepatic cancer (HCC) are largely influenced by sporadic risk factors.^32,33^ Epidemiological studies have indicated that the diet factor is associated with an increasing number of CRC cases,^11,34^ while alcohol consumption is related to HCC cases.^35^ Contaminants in food and drink, arising from reactions during the fermentation process, may account for increasing the risk of developing diseases such as cancer.^36^ Garlic and its bioactive compounds, such as DADS, display antioxidant, cytoprotective, and anti-inflammatory properties, offering protective effects against chemical toxicity.^15^

Here, the resazurin assay revealed that exposing Caco-2 and HepG2 cells to increasing EC concentration decreased cell viability in a concentration- and time-dependent manner. After exposure to 80 mM EC for 24 h, both cell lines had their viability reduced by around 30% compared to that of the negative control. Literature studies have reported a similar decrease in the viability (approximately 30%) of Caco-2 cells exposed to 62.5 mM EC for 24 h.^20^ Similarly, a study involving HepG2 cells has reported that exposure to EC concentrations ranging from 20 to 120 mM for 24 h decreases cell viability in a concentration-dependent manner.^31^

Treating Caco-2 and HepG2 cells with DADS maintained cell viability above 70% at all of the tested DADS concentrations. Results were significant for treatment with 20–120 μM DADS for 24 h and 40–120 μM for 48 h compared to the solvent control. In the literature, different results have been reported. By using the LIVE/DEAD assay, Machado et al.^26^ verified cytotoxicity in HepG2 and HUVEC (human umbilical vein endothelial) cells treated with 50, 100, or 200 μM DADS for 72 h. Kim et al.^23^ demonstrated a dose-dependent decrease in cell viability in both tumor (HT-29, HCT 116, DLD-1) and nontumor (SW 620) colon cell lines treated with 5–100 μM DADS for 24 h.

The toxicity of EC arises from its reactive derivatives and is associated with the carbonyl group, which makes these compounds electrophilic and highly reactive. EC is primarily formed through a reaction between urea and ethanol in an acidic medium. After ingestion, EC is rapidly absorbed by the gastrointestinal tract and metabolized by esterases, undergoing *N*-hydroxylation and oxidation reactions that generate reactive derivatives such as *N*-hydroxyethyl carbamate and vinyl carbamate epoxide.^10^

On the basis of the cell viability results achieved by exposing Caco-2 and HepG2 cells to DADS or EC alone, we selected 10–120 μM DADS and 80 mM EM to proceed with the assays using DADS and EC in combination. EC at 80 mM for 24 h significantly affected cell viability without exceeding 30% cytotoxicity and has been employed in the literature.^19^ In turn, treatment with DADS for 2 or 24 h was chosen because these times represent the time DADS remains unchanged in the cytosol and the time the concentration of DADS metabolites peaks in vivo,^37^ respectively.

By using the resazurin method to analyze cell viability, we observed that pretreatment with DADS for 24 h followed by exposure to EC for 24 h significantly increased cell viability compared to exposure to EC alone. This suggests that DADS can potentially protect against EC-induced cytotoxicity. A study involving HepG2 cells demonstrated that pretreatment with DADS (100 μM) protects against hepatotoxicity induced by 1,3-dichloro-2-propanol (1,3-DCP), an indirect genotoxic food contaminant.^25^ Previous studies have also shown that DADS protects against organ toxicity induced by drugs or toxic substances by inhibiting the activation metabolism of these toxic compounds.^39−41,46,47^

Regarding genotoxic properties, evaluated by the alkaline comet assay, we observed that exposure to EC alone for 4 or 24 h did not damage DNA in Caco-2 or HepG2 cells. This suggests that the selected EC concentration of 80 mM did not promote DNA strand breaks or lesions in these cells.

Although the number of studies evaluating the genotoxicity of EC by using the in vitro comet assay is limited, other assays, such as the micronucleus assay, have evidenced EC-induced chromosomal instability. Shah et al.^41^ observed significantly increased micronuclei percentage in both two-dimensional (2D) and three-dimensional (3D) models of HepG2 cells exposed to 30–50 and 20–50 mM EC, respectively.

Here, DNA damage assessment by the comet assay showed that DNA damage did not increase in Caco-2 or HepG2 cells treated with DADS alone, exposed to EC alone, or exposed to DADS and EC in combination. Literature studies have reported the protective effect of DADS when combined with other genotoxic compounds.^42−44^ Belloir et al.^42^ demonstrated that pretreatment and simultaneous treatment with 5–100 μM DADS for 32 h protects HepG2 cells from DNA damage induced by exposure to various genotoxic agents (e.g., aflatoxin B1, benzopyrene, or *N*-nitrosodimethylamine) for 20 h. In HepG2 cells simultaneously treated with DADS and a genotoxic agent for 24 h, Arranz et al.^43^ observed that 1–5 μM DADS protects against DNA damage induced by *N*-nitrosamine, a compound present in foods.

One of the main mechanisms through which EC exerts toxicity is overproducing ROS, which causes oxidative damage to macromolecules and cellular organelles.^23,44^ By analyzing ROS using the CM-H_2_DCFDA probe, we saw that exposure to EC alone for 24 h increased only ROS levels in Caco-2 cells compared to the solvent control. Although an increase in ROS was observed in both cell types after 4 h of exposure to DADS, studies suggest that short exposures to DADS induce ROS production, possibly due to the neutralization of antioxidant species.^45,46,47^ However, pretreatment of these cells with DADS for 24 h followed by exposure to EC for 24 h reduced ROS levels compared to exposure to EC alone for 24 h. This indicates that DADS can potentially protect against EC-induced ROS in Caco-2 cells. With respect to HepG2 cells, the combined treatment also decreased ROS production compared to exposure to EC alone for 24 h; however, the reduction was not statistically significant compared to that of the solvent control. Kim et al.^25^ reported that DADS exhibits a protective effect on HepG2 cells by reducing oxidative stress induced by a volatile occupational compound. The protective mechanism involves decreased ROS levels, attributed to a reduced expression of the enzyme CYP2E1, which activates the compound. This results in less GSH depletion and reduced MDA production. The authors concluded that DADS exerts its protective effect by down-regulating CYP2E1 expression, a critical enzyme involved in the bioactivation of toxic compounds.

Additionally, Haber et al.^48^ investigated how DADS affects drug-metabolizing enzymes in rat liver and intestine and found that this compound enhances the induction of various cytochrome P450 enzymes. This increases the capacity of these organs to biotransform and eliminate toxins and drugs. The authors attributed this protective effect to the activation of Nrf2 (nuclear factor erythroid 2-related factor 2) signaling pathways, which regulate the expression of genes involved in modulating the antioxidant and detoxification activity of the enzymes.

Shalamitskiy et al.^10^ described that the toxicity of EC and its derivatives is closely related to the carbonyl group, which is highly reactive and can thus cause cellular damage. These compounds increase ROS production, which can induce apoptosis and promote the development of adenomas and hepatocellular carcinoma due to chronic hepatotoxicity. Additionally, ethyl carbamate epoxide forms adducts with DNA, RNA, and proteins, contributing to its carcinogenic effects.^10^ Apoptosis is one of the mechanisms of cell death mediated by EC and occurs primarily via the intrinsic pathway involving mitochondria and the endoplasmic reticulum stress response pathway.^49^ Literature studies have shown that DADS possesses antiapoptotic properties against various chemicals (cadmium, arsenic, and bisphenol A) or natural toxic agents (snake venom).^15^ Our results demonstrate that EC increased the percentage of apoptotic and necrotic cells compared with the negative control in Caco-2 and HepG2 cells. Furthermore, pretreatment with DADS reduced apoptosis-mediated cell death compared to that of cells exposed to EC only.

EC is an indirect genotoxic compound that must be metabolized to exert its toxic effects.^4^ CYP2E1 is the enzyme underlying EC bioactivation.^50^ Conversely, various studies have shown that DADS exerts its protective activity by inhibiting CYP2E1 expression, consequently reducing the metabolic activation of toxic compounds.^15,23,38^ This protective activity occurs through several cellular mechanisms, including decreasing CYP2E1 expression and competing for transcription factors.^38^ Apoptosis is triggered by various physiological and pathological stimuli, such as oxidative damage.^51,52^ Studies with indirect genotoxic compounds bioactivated by CYP2E1 have shown pretreatment with DADS decreases apoptosis levels and increases cell survival by blocking the activation of the JUN N-terminal kinase (JNK) gene, which is associated with oxidative damage and plays a vital role in inducing apoptosis.^38^

In view of the importance of CYP enzymes in bioactivating EC and the results obtained in this study, we must highlight that, although Caco-2 and HepG2 cells are widely used and considered the gold standard in studies on drug absorption, metabolism, and toxicity, they have limitations regarding the expression of cytochrome P450 enzymes.^53,54^ Caco-2 cells exhibit characteristics of differentiated enterocytes, but their CYP expression is low, which prevents them from adequately simulating first-pass intestinal metabolism, the site where many drugs are bioactivated.^55^ HepG2 cells, on the other hand, are a very common model for hepatic studies. Nevertheless, even though they express some phase I and II enzymes, their CYP enzyme expression is insufficient, which results in a limited ability to fully mimic in vivo hepatic metabolism.^56^

Collectively, the results of this study indicate that DADS partially protects against the toxic effects of EC. The beneficial effects of DADS may be related to its ability to reduce oxidative stress by inhibiting CYP2E1. However, further studies of the mechanism of action of DADS against EC should be explored.

## 5 Conclusions

We have highlighted the potential of DADS pretreatment to attenuate the toxic effects of EC in intestinal and hepatic carcinoma cells. The observed reduction in cytotoxicity, apoptosis, and oxidative stress underscores the promising role of DADS in mitigating the adverse impacts of exposure to EC. These findings enhance our understanding of the interplay between DADS and EC and provide insights for further investigations into the intricate mechanisms underlying the combined exposure to them. Ultimately, our study emphasizes that exploring natural compounds like DADS is important and that continued research to optimize their therapeutic potential is essential.
